# Prevalence of carbapenemase genes among multidrug-resistant *Pseudomonas aeruginosa* isolates from tertiary care centers in Southern Thailand

**DOI:** 10.15537/smj.2022.43.9.20220219

**Published:** 2022-09

**Authors:** Phanvasri Saengsuwan, Narongdet Kositpantawong, Soontara Kawila, Wichien Patugkaro, Chonticha Romyasamit

**Affiliations:** *From the Department of Biomedical Sciences and Biomedical Engineering (Saengsuwan), From the Department of Internal Medicine (Kositpantawong), from the Department of Pathology (Kawila), Microbiology Unit, Faculty of Medicine, Prince of Songkla University; from the Microbiology Clinical Pathology Division (Patugkaro), Hat Yai Hospital, Hat Yai; and from the School of Allied Health Sciences (Romyasamit), Walailak University, Walailak, Thailand.*

**Keywords:** carbapenems, epidemiology, multidrug resistance, multiplex PCR, pulsed field gel electrophoresis, *Pseudomonas aeruginosa*

## Abstract

**Objectives:**

To assess the prevalence of carbapenemase genes among multidrug-resistant *Pseudomonas aeruginosa* (*P. aeruginosa*) isolates from tertiary care centers in Southern Thailand.

**Methods:**

The prevalence of carbapenemase genes in *P. aeruginosa* isolates collected from patients hospitalized between 2015-2017 in 2 tertiary care hospitals in Songkhla Province, Southern Thailand, was investigated. Standard laboratory procedures were followed and disk diffusion test was used for bacterial identification and susceptibility evaluations. Carbapenemase genes were detected using multiplex polymerase chain reaction (PCR) and genotyping by pulsed field gel electrophoresis.

**Results:**

Among the 289 *P. aeruginosa* isolates, 55% was from sputum, 19.4% was from urine, and 8% was from secretions. The prevalence was 55.7% in carbapenem-resistant multidrug-resistant *P. aeruginosa* (CR-MDR-PA) and 39.4% in multidrug-resistant *P. aeruginosa* (MDR-PA). Resistance to imipenem, meropenem, gentamicin, and ceftazidime ranged from 50-60%, and amikacin was the most effective antibiotic (38.4%). The carbapenemase genes *bla*_VIM_ (27.7%), *bla*_IMP_ (23.9%), and *bla*_OXA48_ (4.8%) were detected; however, *bla*_SPM_ and *bla*_BIC_ were not detected in any of the isolates. Pulsed field gel electrophoresis analysis revealed clonal diversity among 17 CR-MDR-PA strains.

**Conclusion:**

A high percentage of CR-MDR-PA carries carbapenemase genes in our area; therefore, more emphasis on and application of molecular techniques for infection prevention and control may provide useful insights on disease epidemiology.

Multidrug-resistant *Pseudomonas aeruginosa* (MDR-PA) has become a worldwide health problem because it exhibits broad resistance to carbapenems, including “last-line” carbapenems.^1^ The prevalence of MDR-PA and extensively drug-resistant *P. aeruginosa* (XDR-PA) producing carbapenemase has been increasing. One mechanism of this resistance is the degradation of carbapenems by enzyme lactamases such as carbapenemase. Carbapenemases are classified into 3 molecular molecules: class A metallo-β-lactamases (MBLs), which mostly include the enzyme *Klebsiella pneumoniae* carbapenemase gene (*bla*_KPC_); class B MBLs, such as Verona integron-encoded MBL (*bla*_VIM_), and *bla*_IMP_ types, which were subsequently mutated to New Delhi MBL 1 (*bla*_NDM-1_); and class D (oxacillinases or *bla*_OXA_), which is produced by *P. aeruginosa*. The prevalence of *P. aeruginosa* infection has increased in the last decade, particularly in healthcare settings, and has been recognized by the Centers of Disease Control and Prevention (CDC).^2^

The National Antimicrobial Resistance Surveillance Center reported that the incidence of *P. aeruginosa* infections in hospitals in Thailand has dramatically increased in the past 15 years. In particular, the Department of Medical Sciences found that the incidence of imipenem (IMP)-resistant *P. aeruginosa* in the country increased from 14% in 2000 to 47% in 2015, resulting in higher morbidity rates.^3,4^

The transmission of carbapenemase genes among carbapenem-resistant *P. aeruginosa* (CR-PA) isolates should be carefully considered because many of these genes are carried by plasmids and are easily transferable. Phenotypic techniques, such as the modified Hodge test, for the in vitro identification of carbapenemase production are not very sensitive and specific.^5^ Carbapenemase detection may be based on the inhibitory properties of several molecules. Furthermore, although the molecular detection of carbapenemase genes is a viable alternative, it is still seldom used because of its high cost and requirement of data-interpretation expertise.^6^

Presently, carbapenem resistance in *P. aeruginosa* typically results from the formation of class B carbapenemase and has led to a global epidemic of *P. aeruginosa* infection.^7,8^ However, the Clinical Laboratory Standards Institute (CLSI) has not yet established a standardized method for screening and assaying carbapenemase.^9,10^ Although several methods are available to diagnose an infection and determine *P. aeruginosa* resistance, the most accepted is in vitro culture. Susceptibility tests are the gold standard, but they are labor-intensive because only one drug concentration can be tested in each tube. Therefore, understanding the potential resistance mechanisms of *P. aeruginosa* is imperative to select the effective antimicrobial agents. Thus, this study aimed to examine the prevalence of carbapenemase genes in *P. aeruginosa* isolates from patients admitted to tertiary care hospitals in Southern Thailand by using the multiplex polymerase chain reaction (mPCR) technique, investigate the antimicrobial susceptibility profile, and then identify any strains that could potentially cause an outbreak by using pulsed field gel electrophoresis (PFGE).

## Methods

This retrospective study reviewed the data regarding *P. aeruginosa* isolates of patients admitted to 2 tertiary care hospitals in Songkhla, Thailand, between August 2015 and March 2017. The study included all consecutive nonduplicate isolates of *P. aeruginosa* (n=289) resistant to meropenem (MEM) or IMP (based on disk diffusion test findings) from various clinical samples (sputum, blood, urine, secretions [penrose drain, bronchial wash, percutaneous nephrostomy, corneal ulcer, bile, and pleural fluid], pus, tissue, and catheter tip). Bacteria were identified at the hospital’s microbiology laboratory using conventional biochemical tests in accordance with the 2015 CLSI guidelines. All *P. aeruginosa* isolates were stored in 20% glycerol at -80°C until further tests were carried out.

Drug susceptibility was tested and interpreted using disk diffusion test according to the 2015 CLSI guidelines.^9^ Each disk (Becton Dickinson, Heidelberg, Germany) contained amikacin (AK, 30 µg), ceftazidime (CAZ, 30 µg), ciprofloxacin (CIP, 5 µg), colistin (DA, 10 µg), gentamicin (GM, 10 µg), IMP (10 µg), MEM (10 µg), norfloxacin (NOR, 10 µg), cefoperazone/sulbactam (Sulperazone [SPZ], 75/30 µg), ceftriaxone (CRO, 30 µg), ertapenem (ERT, 10 µg), levofloxacin (LVX, 5 µg), sitafloxacin (STFX, 5 µg), cefotaxime (CTX, 30 µg), or piperacillin/tazobactam (Tazocin) (TZP, 100 µg).^11^ According to the CDC criteria, a strain resistant to at least one agent in 3 or more antipseudomonal antimicrobial categories was considered MDR-PA that is resistant to at least one agent in all but 2 or fewer antipseudomonal antimicrobial categories was considered XDR-PA, and that resistant to all agents in all antipseudomonal antimicrobial categories was considered pandrug-resistant *P. aeruginosa* (PDR-PA).^12^

The GF-1 bacterial DNA extraction kit (Vivantis Technologies Sdn. Bhd., Selangor Darul Ehsan, Malaysia) was used for isolating genomic DNA according to the manufacturer’s instructions, and a spectrophotometer for quantifying DNA concentrations at an absorbance of 260 nm (A260). Deoxyribonucleic acid purity was calculated from the A260/A280 ratio, and DNA quality was evaluated using agarose gel electrophoresis.

The following primers and conditions for mPCR for carbapenemase gene amplification (Table 1) were used, as previously described.^13,14^ An expert from of the Centre for Genetics Consultation and Cancer Screening, 108 Military Central Hospital, Hanoi, Vietnam, and the staff provided template DNA from isolates showing carbapenemase production (*bla*_AIM_, *bla*_BIC_, *bla*_DIM_, *bla*_KPC_, *bla*_IMP_, *bla*_OXA48_, *bla*_SPM_, and *bla*_VIM_ genes). The PCR products (1st BASE DNA Sequencing Services, Selangor, Malaysia) were sequenced and then the sequence similarity was determined using the Basic Local Alignment Search Tool through the National Center for Biotechnology Information database.

**Table 1:** - List of primers used for amplifying carbapenemase genes.

Primer	Sequence (5′→3′)	Genes	Product sizes (bp)
*IMP-F*	*GGAATAGAGTGGCTTAAYTCTC*	*bla*_*IMP*_	232
*IMP-R*	*GGTTTAAYAAAACAACCACC*		
*SPM-F*	*AAAATCTGGGTACGCAAACG*	*bla*_*SPM*_	271
*SPM-R*	*ACATTATCCGCTGGAACAGG*		
*AIM-F*	*CTGAAGGTGTACGGAAACAC*	*bla*_*AIM*_	322
*AIM-R*	*GTTCGGCCACCTCGAATTG*		
*VIM-F*	*GATGGTGTTTGGTCGCATA*	*bla*_*VIM*_	390
*VIM-R*	*CGAATGCGCAGCACCAG*		
*OXA-F*	*GCGTGGTTAAGGATGAACAC*	*bla*_*OXA-48*_	438
*OXA-R*	*CATCAAGTTCAACCCAACCG*		
*GIM-F*	*TCGACACACCTTGGTCTGAA*	*bla*_*GIM*_	477
*GIM-R*	*AACTTCCAACTTTGCCATGC*		
*BIC-F*	*TATGCAGCTCCTTTAAGGGC*	*bla*_*BIC*_	537
*BIC-R*	*TCAATTGGCGGTGCCGTACAC*		
*SIM-F*	*TACAAGGGATTCGGCATCG*	*bla*_*SIM*_	570
*SIM-R*	*TAATGGCCTGTTCCCATGTG*		
*NDM-F*	*GGTTTGGCGATCTGGTTTTC*	*bla*_*NDM*_	621
*NDM-R*	*CGGAATGGCTCATCACGATC*		
*DIM-F*	*GCTTGTCTTCGCTTGCTAACG*	*bla*_*DIM*_	699
*DIM-R*	*CGTTCGGCTGGATTGATTTG*		
*KPC-Fm*	*CGTCTAGTTCTGCTGTCTTG*	*bla*_*KPC*_	798
*KPC-Rm*	*CTTGTCATCCTTGTTAGGCG*		232

Conforming to the principles of the Declaration of Helsinki, this study was approved by the Ethics Committees of the Faculty of Medicine, Prince of Songkla University (REC-58-183-04-8), Songkla, Thiland, and the Ethics Committee of Hatyai Hospital (Protocol No.: 61/58), Hatyai, Thailand. Considering that the samples were collected from patients as part of standard diagnostic care, informed consent was not required for this study. An expert from the Centre for Genetics Consultation and Cancer Screening, 108 Military Central Hospital granted material transfer agreement for the transportation of positive controls.

Pulsed field gel electrophoresis was carried out as previously described by Pfaller et al^15^ and modified by Seifer et al.^16^ Briefly, an overnight culture of *P. aeruginosa* with an optical density (OD600) of 0.5 in Luria Bertani broth (Merck KGaA, Darmstadt, Germany) was incubated in 100 mM Tris pH 7.2 buffer containing 100 mM ethylenediaminetetraacetic acid (EDTA; Amresco, Solon, Ohio, USA), 20 mM NaCl (Amresco, Solon, Ohio, USA), and 0.5 mg/ml concentration of proteinase K (Amresco, Solon, Ohio, USA) at 55°C for 10 minutes. Subsequently, an equal volume of 2% UltraPureTM LMP agarose (Invitrogen, Carlsbad, CA, USA) was added, and the solution was placed in a mold to form a solid plug, which was then incubated with cell lysis buffer (50 mM Tris pH 8.0, 100 mM EDTA, 0.1% sodium dodecyl sulfate, 1.0% sarcosine [Amresco, Solon, Ohio, USA], and 0.5 mg/ml concentration of proteinase K) at 55°C for 2 hours. The agarose plug was treated with 10 U of XbaI (Fermentas, USA).^15^ The DNA was separated by PFGE using a CHEF-DR III system (Bio-Rad Laboratories, Hercules, CA, USA). Running conditions were 21 hours at 14°C, with an initial switching time of one second and final time of 30 seconds, at 6 V/cm. Band patterns were analyzed using the BioNumerics 7.0 software (Applied Maths, St-Martens-Latem, Belgium) and interpreted according to the Tenover Interpretive Criteria.^17^

### Statistical analysis

All statistical data were analyzed using the Statistical Package for the Social Sciences, version 23.0 (IBM Corp., Armonk, NY, USA). Categorical variables are reported as numbers and percentages, and each variable was examined by univariate analysis. Multinomial logistic regression was used for calculating odds ratios (ORs), 95% confidence intervals (CIs), and *p*-values and for further analyzing variables with *p*<0.05 on univariate analysis. All tests were 2-tailed, and a *p*-value of <0.05 was considered significant.

To determine factors associated with carbapenem resistance, *P. aeruginosa* isolates were categorized as follows: carbapenem-resistant multidrug-resistant *P. aeruginosa* (CR-MDR-PA), CR-PA, and control (carbapenem-susceptible *P. aeruginosa* [CS-PA]).

## Results

A total of 289 nonduplicate *P. aeruginosa* isolates were submitted to the microbiology laboratory. Table 2 lists the demographic data of patients infected with CS-PA, MDR-PA, and CR-MDR-PA isolates. Out of the 289 isolates, 178 (61.6%) were from male patients and 111 (38.4%) from female patients. The median age was 61.0 years, and the mean hospitalization period was 36.8 days. Sputum was the most common specimen (55%), followed by urine (15.2%), secretion (8.0%), and pus (5.5%). The most common underlying disease was pulmonary disorders (12.5%), followed by gastrointestinal diseases (10.7%), infectious diseases (6.9%), malignancies (6.6%), and neurological diseases (6.2%). Pulmonary and extrapulmonary isolates (blood, tissue, pleural fluid, and abscess) showed no statistically significant differences. Further epidemiological data are presented in Table 2.

**Table 2:** - Characteristics of clinical specimens of patients with Pseudomonas aeruginosa infections.

Variables			CS PA (n=14)			MDR-PA (n=114)		CR-MDR-PA (n=161)	Total (N=289)		
***Gender***											
Male			10 (3.5)			70 (24.2)		98 (33.9)	178 (61.6)		
Female			4 (1.4)			44 (15.2)		63 (21.8)	111 (38.4)		
***Underlying condition***											
Pulmonary disease			2 (0.7)			14 (4.8)		20 (6.9)	36 (12.5)		
Gastrointestinal disease			1 (0.3)			14 (4.8)		16 (5.5)	31 (10.7)		
Infectious disease			1 (0.3)			15 (5.2)		4 (1.4)	20 (6.9)		
Malignancy			2 (0.7)			10 (3.5)		7 (2.4)	19 (6.6)		
Neurologic disease			1 (0.3)			13 (4.5)		4 (1.4)	18 (6.2)		
Genitourinary disease			1 (0.3)			7 (2.4)		8 (2.8)	16 (5.5)		
Cardiovascular disease			2 (0.7)			9 (3.1)		5 (1.7)	16 (5.5)		
Bone			0 (0.0)			9 (3.1)		7 (2.4)	16 (5.5)		
Hematoma			0 (0.0)			8 (2.8)		4 (1.4)	12 (4.2)		
***Specimens***											
Sputum			11 (3.8)			70 (24.2)		78 (27.0)	159 (55.0)		
Urine			2 (0.7)			10 (3.5)		44 (15.2)	56 (19.4)		
Secretions*			0 (0.0)			8 (2.8)		15 (5.2)	23 (8.0)		
Pus			0 (0.0)			8 (2.8)		8 (2.8)	16 (5.5)		
Tissue			0 (0.0)			9 (3.1)		6 (2.1)	15 (5.2)		
Blood			0 (0.0)			7 (2.4)		7 (2.4)	14 (4.8)		
Catheter			1 (0.3)			2 (0.7)		3 (1.0)	6 (2.1)		
ICU			3 (1.0)			22 (7.6)		26 (9.0)	51 (17.6)		
Non-ICU			11 (3.8)			92 (31.8)		161 (46.7)	238 (82.4)		
Hat Yai Hospital			2 (0.7)			7 (2.4)		79 (27.3)	88 (30.4)		
Songklanagarind Hospital			12 (4.2)			107 (37.0)		82 (28.4)	201 (69.6)		
***Grouped by age***											
0-12 years			2 (0.7)			18 (6.2)		17 (5.9)	37 (12.8)		
13-24 years			0 (0.0)			4 (1.4)		7 (2.4)	11 (3.8)		
25-64 years			4 (1.4)			52 (18.0)		58 (20.1)	114 (39.4)		
≥65 years			8 (2.8)			40 (13.8)		79 (27.3)	127 (43.9)		

Values are presented as a number and precentage (%). *Penrose drain, bronchial wash, percutaneous nephrostomy, corneal ulcer, bile, and pleural fluid. CS-PA: carbapenem-susceptible *Pseudomonas aeruginosa*, MDR-PA: multidrug-resistant *Pseudomonas aeruginosa*, CR-MDR-PA: carbapenem-resistant multidrug-resistant *Pseudomonas aeruginosa*, ICU: intensive care unit

The incidence of CR-MDR-PA was the highest, and it was detected mostly from non- wards (46.7%), followed by ICU (9%), medical wards (18.9%), and all other wards (15.4%). However, the infection site had no statistically significant effect on the susceptibility to infection with CR-MDR-PA and MDR-PA.

Multidrug-resistant *P. aeruginosa* and CR-MDR-PA were more frequently found in Songklanagarind Hospital; their incidence was 15.4 times and one time higher than that in Hat Yai Hospital, with statistical significance (*p*<0.05). Patient characteristics were generally similar among patients infected with MDR-PA/CS-PA, CR-MDR-PA/CS-PA, and MDR-PA/CR-MDR-PA. However, patients aged 0-12 years with MDR-PA infection were 72% more likely to be diagnosed with MDR-PA infection than those aged >65 years with CR-MDR-PA infection (95% CI: [1-0.285], *p*<0.05). Further, MDR-PA infections were more common in patients aged 25-64 years than in those aged >65 years with CR-MDR-PA infection (95% CI: [1-0.309], *p*<0.05; Table 3).

**Table 3:** - Univariate analysis of risk factors for carbapenemase-encoding Pseudonomas aeruginosa infections.

Variables	Univariate analysis					
	MDR-PA/CS-PA	*P*-value	CR-MDR/CS-PA	*P*-value	MDR-PA/CR-MDR-PA	*P*-value
***Gender***						
Male	0.636	0.515	0.349	0.133	0.549	0.078
Female						
***Underlying diseases***						
Pulmonary disease	3.261	0.302	5.174	0.149	1.587	0.499
Gastrointestinal disease	4.076	0.306	4.885	0.249	1.199	0.795
Infectious disease	7.375	0.169	2.839	0.495	0.385	0.247
Malignancy	1.304	0.829	1.088	0.947	0.834	0.813
Neurologic disease	6.583	0.162	1.865	0.66	0.283	0.136
Genitourinary disease	1.24	0.88	2.297	0.555	1.853	0.43
Cardiovascular disease	1.792	0.618	0.648	0.723	0.362	0.23
Bone	-	-	-	-	0.866	0.859
Hematoma	-	-	-	-	0.867	0.87
***Specimens***						
Sputum	4.201	0.339	2.516	0.517	0.599	0.661
Urine	2.748	0.545	5.193	0.297	1.89	0.6
Secretions*	-	-	-	-	1.384	0.794
Pus	-	-	-	-	0.62	0.714
Tissue	-	-	-	-	0.713	0.792
Blood	-	-	-	-	0.464	0.563
Catheter						
ICU	0.632	0.591	0.665	0.636	1.051	0.905
Non-ICU						
Hat Yai Hospital	1.335	0.815	23.023	0.009	17.247	0
Songklanagarind Hospital						
***Age***						
0-12 years	1.742	0.608	0.497	0.519	0.285	0.023
13-24 years	-	-	-	-	0.544	0.44
25-64 years	2.172	0.282	0.671	0.584	0.309	0.002
≥65 years						

Values are presented as an odds ratio (OR). *Penrose drain, bronchial wash, percutaneous nephrostomy, corneal ulcer, bile, and pleural fluid. CS-PA: carbapenem-susceptible *Pseudonomas aeruginosa*, MDR-PA: multidrug-resistant *Pseudonomas aeruginosa*, CR-MDR-PA: carbapenem-resistant multidrug-resistant *Pseudonomas aeruginosa*, ICU: intensive care unit

Table 4 shows the antimicrobial susceptibility of the isolates. Among the isolates, 57.8% were resistant to IMP, 51.9% were resistant to GM, and 49.5% were resistant to MEM. The isolates were mostly susceptible to AK (38.4%), TZP (36.3%), and GM (35.3%). Of the 289 isolates, 55.7% were CR-MDR-PA, 39.4% were MDR-PA, and none were PDR-PA.

**Table 4:** - Antimicrobial susceptibility among 289 clinical isolates of Pseudomonas aeruginosa.

Mode of action	Class	Antimicrobial agents	CS-MDR-PA (n=14)		MDR-PA (n=114)		CR-MDR-PA (n=161)		Total	
			Sensitive	Resistant	Sensitive	Resistant	Sensitive	Resistant	Sensitive	Resistant
Protein synthesis (30S ribosomal subunit)	Aminoglycosides	Amikacin	11 (78.6)	2 (14.3)	99 (86.8)	11 (9.6)	1 (0.6)	128 (79.5)	111 (38.4)	141 (48.8)
		Gentamicin	8 (57.1)	5 (35.7)	94 (82.5)	17 (14.9)	0 (0.0)	128 (79.5)	102 (35.3)	150 (51.9)
	β-lactams (cephalosporins), 3rd generation	Cefotaxime	0 (0.0)	0 (0.0)	0 (0.0)	0 (0.0)	0 (0.0)	6 (3.7)	0 (0.0)	6 (2.1)
		Ceftazidime	3 (21.4)	8 (57.1)	69 (60.5)	15 (13.2)	2 (1.2)	117 (72.7)	74 (25.6)	140 (48.4)
		Ceftriaxone	0 (0.0)	0 (0.0)	3 (2.6)	1 (0.9)	0 (0.0)	67 (41.6)	3 (1.0)	68 (23.5)
		Cefoperazone/sulbactam	5 (35.7)	3 (21.4)	66 (57.9)	21 (18.4)	18 (11.2)	102 (63.4)	89 (30.8)	126 (43.6)
Cell wall synthesis	β-lactams (carbapenem)	Imipenem	13 (92.9)	0 (0.0)	4 (3.5)	102 (89.5)	2 (1.2)	65 (40.4)	19 (6.6)	167 (57.8)
		Meropenem	13 (92.9)	0 (0.0)	21 (18.4)	76 (66.7)	0 (0.0)	67 (41.6)	34 (11.8)	143 (49.5)
		Ertapenem	1 (7.1)	0 (0.0)	0 (0.0)	2 (1.8)	0 (0.0)	31 (19.3)	1 (0.3)	33 (11.4)
	Combinations: piperacillin (β-lactams) and tazobactam (β-lactamase inhibitors)	Piperacillin/tazobactam	2 (14.3)	3 (21.4)	69 (60.5)	16 (14.0)	34 (21.1)	101 (62.7)	105 (36.3)	120 (41.5)
DNA gyrase	Fluoroquinolone	Levofloxacin	0 (0.0)	1 (7.1)	5 (4.4)	2 (1.8)	1 (0.6)	77 (47.8)	6 (2.1)	80 (27.7)
		Ciprofloxacin	7 (50.0)	4 (28.6)	81 (71.1)	26 (22.8)	0 (0.0)	61 (37.9)	88 (30.4)	91 (31.5)
		Norfloxacin	0 (0.0)	2 (14.3)	9 (7.9)	4 (3.5)	0 (0.0)	17 (10.6)	9 (3.1)	23 (8.0)
		Sitafloxacin	0 (0.0)	0 (0.0)	0 (0.0)	0 (0.0)	0 (0.0)	4 (2.5)	0 (0.0)	4 (1.4)
Cell membrane	Lipopeptides	Colistin	2 (14.3)	0 (0.0)	19 (16.7)	0 (0.0)	60 (37.3)	0 (0.0)	81 (28.0)	0 (0.0)

CS-MDR-PA: carbapenem-susceptible multidrug-resistant *Pseudomonas aeruginosa*, MDR-PA: multidrug-resistant *Pseudomonas aeruginosa*, CR-MDR-PA: carbapenem-resistant multidrug-resistant *Pseudomonas aeruginosa*

The incidence of *P. aeruginosa* infections was the highest (11.8%) in December 2016 and lowest (0.7%) in December 2015 (Appendix 1).

The most common carbapenemase gene in all *P. aeruginosa* isolates was *bla*_VIM_ (27.7%), followed by *bla*_IMP_ (69, 23.9%), *bla*_DIM_ (54, 18.7%), and others-*bla*_OXA_ (34, 11.8%), *bla*_AIM_ (30, 10.4%), *bla*_NDM_ (9, 3.1%), *bla*_GIM_ (5, 1.7%), *bla*_KPC_ (4, 1.4%), and *bla*_SIM_ (4, 1.4%). Conversely, *bla*_SPM_ and *bla*_BIC_ were not found (Appendix 2).

**Figure 1:**
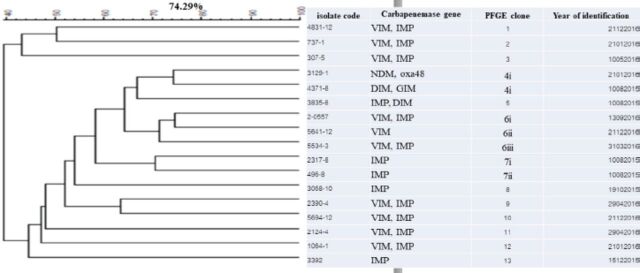
- Dendrogram of *Pseudomonas aeruginosa* isolated from CR-MDR-PA in tertiary care hospitals of Songkhla Province. The PFGE profiles were analyzed using the BioNumerics software, the similarity of band patterns was computed using Pearson’s correlation coefficient, and a dendrogram was generated using an unweighted pair group of the arithmetic mean approach. The isolates were clustered into groups with >70% similarity. The scale represents the percentage of similarity. CR-MDR-PA: carbapenem-resistant multidrug-resistant *Pseudomonas aeruginosa*, PFGE: pulsed field gel electrophoresis

The dendrogram for genetic similarity was generated using the macrorestriction profile, and data from the 17 most common *P. aeruginosa* isolates are summarized in Figure 1. All these 17 isolates, which were divided into 13 different genotypes, underwent PFGE. All isolates collected from hospital showed high genetic variation, and 2 were MDR-PA. Other isolates were found from different sites, and they belonged to 5 genotypes. All 17 *bla*_IMP_-positive isolates belonged to 11 clusters, but one cluster, which was linked to 2 isolates from ICU-obtained sputum samples, had *bla*_NDM_, *bla*_oxa48_, *bla*_DIM_, and *bla*_GIM_.

## Discussion

*Pseudomonas aeruginosa* is a common cause of nosocomial infections and results in high mortality rates.^18^ This study found that MDR-PA isolates were a common cause of infection caused by CR-PA during the study period. This finding may be explained by the heavy use of antibiotics in patients, thereby, increasing the emergence of the XDR phenotype. The incidence of MDR-PA (55.7%) in this study is similar to that in other studies, such as 56% in Egypt.^17^ However, in our study, *P. aeruginosa* infections mostly occurred in the respiratory tract, followed by the urinary tract, and secretions. This result is similar to the 57.3% rate of *P. aeruginosa* infection reported by Swathirajan et al^19^ in China; this rate was previously described in infected patients with cystic fibrosis.^20^

The current study revealed that *P. aeruginosa* infection was a major cause of complications, particularly pneumonia, in children (17, 5.9%) and older patients (58, 20.1%). Several reasons for why these patients suffered from this infection type were determined. Our findings are consistent with those of another study carried out at Single University Hospital Center in Germany. According to this previous study, *P. aeruginosa* caused most cases of pneumonia, with a mean patient age of 68.1±12.8 years (113 [67.3%] males and 55 [32.7%] females).^21^

Carbapenem-resistant MDR-PA infection most commonly occurred in the non-intensive care unit (ICU) wards, with an incidence rate of 46.7%. Bhatt et al^22^ reported that the incidence of infections caused by resistant *P. aeruginosa* was 54.9% in the burn unit in India, consistent with our results. In our study, all resistant isolates were obtained between 2015-2017, demonstrating a 2-fold increase in the incidence of *P. aeruginosa* infection at Songklanagarind Hospital compared with that in Hat Yai Hospital, Thailand. This result could be explained by the number of available beds, which has an impact on nosocomial infection outbreak.^23^

Susceptibility tests showed that 161 CR-MDR-PA isolates tested against 16 antimicrobial agents were highly resistant to AK, GM, CAZ, and TZP. Several isolates were also resistant to LVX, MEM, and IMP. Resistance to quinolones (CIP and LVX) ranged from 30-50% and that to NOR (10.6%) and STFX (2.5%).

As observed, CR-MDR-PA was resistant to various antimicrobial agents, except DA. CR-MDR-PA was previously reported to be susceptible to TZP.^24,25^ Furthermore, MBL-producing PA is less sensitive to aztreonam, possibly because of the different resistance mechanisms in *P. aeruginosa*.

In addition to MDR-PA strains, nearly 90% of the isolates were highly resistant to carbapenem. These isolates were completely resistant to IMP and MEM but were susceptible to AK (86.8%), GM (82.5%), and CIP (71.1%). Therefore, with proper use, AK can still be an effective treatment drug against *P. aeruginosa* infection.

According to previous studies in Thailand, *bla*_VIM_ is the most common carbapenemase gene detected in *P. aeruginosa* and is widely prevalent in the country.^26^ In our study, *bla*_VIM_ (n=80, 22.7%) was the most common, contrary to the result (12.5%) from the study of the clinical CR-PA isolates from Phramongkutklao Hospital, Thailand.^27^

Surprisingly, *bla*_DIM_ was found in our region (18.7%). Likewise, *bla*_DIM_ was found in 5 out of 200 clinical isolates of *P. aeruginosa* (2.5%) in India, lower than that observed in our study.^28^ The high prevalence of *bla*_DIM_ may be explained by transgenic gene resistance. These findings clearly demonstrate that other determinants may also be implicated in the prevalence of antibiotic-resistance genes.

Pulsed field gel electrophoresis analysis revealed that *P. aeruginosa* harboring *bla*_VIM_ is the clonally predominant genotype. This major clonality (70%) suggests that cross-transmission is an important mechanism of dissemination, causing high resistance levels among *P. aeruginosa* isolates. Therefore, continuous surveillance and improved infection control strategies are needed to reduce cross-infection, particularly when majority of the carbapenemase genes are on high mobility.

### Study limitations

First, isolates from only 2 hospitals were examined, with most of them being collected from a single institution, thereby limiting the generalization of the results. Second, no DNA sequencing (whole-genome sequencing or at least amplicon sequencing) was carried out to determine the carbapenemase variants that are distributed in our geographical region. Third, the data were retrospectively collected; hence, patient data such as comorbidities and other clinical information could not be collected. Finally, antimicrobial resistance mechanisms and their potential interactions with virulence factor genes were not investigated. More studies will be carried out in the future by multilocus sequencing to elucidate the population genetics of CR-PA isolates from Thailand, and whole-genome sequences will be utilized to explore more epidemiological features of carbapenemase-producing *P. aeruginosa* in clinical studies.

In conclusion, the clinical isolates of CR-MDR-PA are highly prevalent in Southern Thailand. Several carbapenemase genes, particularly *bla*_VIM_ and *bla*_IMP_, are present; these genes are associated with genotyping demonstrated in the endemic spread of genetically closely related strains. Our results provide a new possibility for utilizing molecular techniques to control antimicrobial resistance in our region, and prudent antibiotic administration may help limit infection spread. These findings may aid in the improvement of infection control and clinical treatment protocols to lessen the impact of these infections on hospitalized patients.

## Figures and Tables

**Appendix 1:**
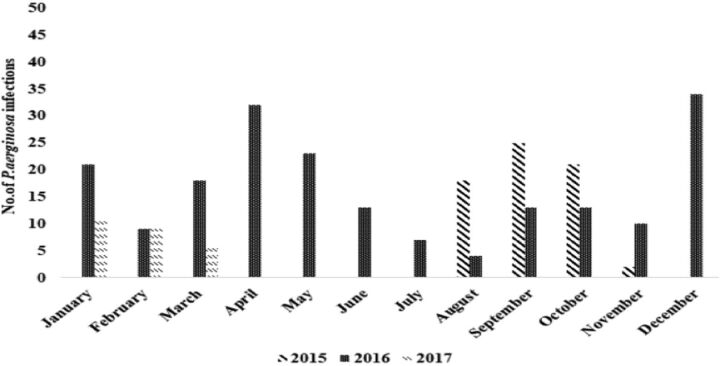
- The occurrence of 289 *Pseudomonas aeruginosa (P. aeruginosa*) isolated from various specimens in tertiary care hospitals of Songkhla Province between August 2015 and March 2017.

**Appendix 2:**
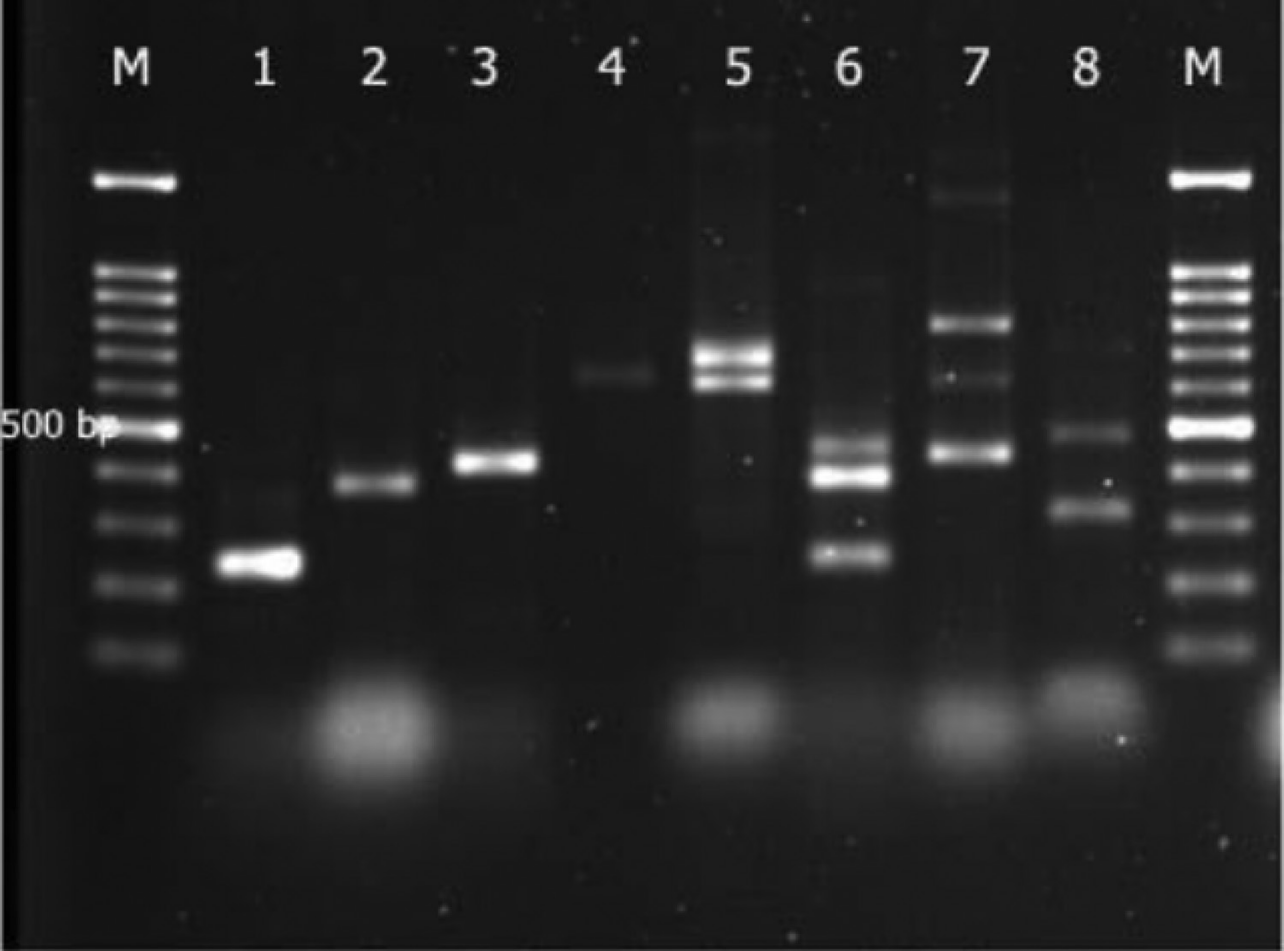
- Multiplex PCR-based genotypic characterization of *Pseudomonas aeruginosa (P. aeruginosa*) isolates recovered from clinicsl isolates. Lane M: 100 bp molecular size marker, Lane 1-5: clinical P. *aeruginosa* isolates showing band at 232 bp denoting *bla*_IMP_, *bla*_VIM_, *bla*_OXA48_, *bla*_NDM_, *bla*_DIM_, and *bla*_SIM_ gene band at 390, 438, 621, 699, and 570 bp, Lane 6-8: multiplex PCR were control strains
